# Mitochondrial respiratory chain deficiency correlates with the severity of neuropathology in sporadic Creutzfeldt-Jakob disease

**DOI:** 10.1186/s40478-020-00915-8

**Published:** 2020-04-16

**Authors:** Irene H. Flønes, Gerda Ricken, Sigrid Klotz, Alexandra Lang, Thomas Ströbel, Christian Dölle, Gabor G. Kovacs, Charalampos Tzoulis

**Affiliations:** 1 Neuro-SysMed, Department of Neurology, Haukeland University Hospital, 5021 Bergen, Norway; 2 Department of Clinical Medicine, University of Bergen, Pb 7804, 5020 Bergen, Norway; 3 Institute of Neurology, Medical University of Vienna, Vienna, Austria; 4 Tanz Centre for Research in Neurodegenerative Disease, University of Toronto, Toronto, Ontario Canada; 5 Laboratory Medicine Program, University Health Network, Toronto, Canada

**Keywords:** Mitochondria, Respiratory chain, Prion, Neurodegeneration, PrP

## Abstract

Mitochondrial dysfunction has been implicated in multiple neurodegenerative diseases but remains largely unexplored in Creutzfeldt-Jakob disease. Here, we characterize the mitochondrial respiratory chain at the individual neuron level in the MM1 and VV2 common molecular subtypes of sporadic Creutzfeldt-Jakob disease. Moreover, we investigate the associations between the mitochondrial respiratory chain and neuropathological markers of the disease.

Brain tissue from individuals with sporadic Creutzfeldt-Jakob disease and age-matched controls were obtained from the brain collection of the Austrian Creutzfeldt-Jakob Surveillance. The mitochondrial respiratory chain was studied through a dichotomous approach of immunoreactivities in the temporal cortex and the hippocampal subregions of CA4 and CA3.

We show that profound deficiency of all mitochondrial respiratory complexes (I-V) occurs in neurons of the severely affected temporal cortex of patients with Creutzfeldt-Jakob disease. This deficiency correlates strongly with the severity of neuropathological changes, including vacuolation of the neuropil, gliosis and disease associated prion protein load. Respiratory chain deficiency is less pronounced in hippocampal CA4 and CA3 regions compared to the temporal cortex. In both areas respiratory chain deficiency shows a predilection for the MM1 molecular subtype of Creutzfeldt-Jakob disease.

Our findings indicate that aberrant mitochondrial respiration could be involved early in the pathogenesis of sporadic Creutzfeldt-Jakob disease and contributes to neuronal death, most likely via ATP depletion. Based on these results, we propose that the restricted MRI diffusion profile seen in the brain of patients with sporadic Creutzfeldt-Jakob disease might reflect cytotoxic changes due to neuronal respiratory chain failure and ATP loss.

## Introduction

Sporadic Creutzfeldt-Jakob disease (sCJD) is a severe and lethal neurodegenerative disease that commonly manifests between the sixth and seventh decade of life with an overall incidence of 1–2 /million / year [1]. While it is most often recognized by rapidly progressive dementia and myoclonus, it has a broad spectrum of clinical presentations including ataxia, visual disturbances, tremor and other movement disorders. The neuropathology of sCJD is characterized by neuronal loss, vacuolation of the neuropil, reactive gliosis and disease-associated prion protein (PrP^sc^) deposits [2]. PrP^sc^ are pathological aggregations of prion protein (PrP^c^), a naturally occurring protein of unclear physiological function encoded by the *PRNP* gene. sCJD differs from other neurodegenerative proteinopathies in that self-aggregating PrP^sc^ is considered to be the primary disease-causing event, and that it is transmissible through nerve to nerve contact [1]. PrP^sc^ aggregation is disease specific and is found both in subtle synaptic and perineuronal accumulations and more granular, perivacuolar and plaque-like accumulations depending on disease subtype [2]. PrP^sc^ has been shown to exist in molecular subtypes that show differences in size and degree of glycosylation, degree of protease resistance, aggregation state and conformational stability; namely PrP^sc^ type 1 and 2 [3, 4].

Clinical and pathological features of sCJD are strongly influenced by the molecular subtype of PrP^sc^ and the genotype of codon 129 of the *PRNP* gene, coding for either methionine or valine [2, 5]. Individuals who are homozygous for the methionine codon (MM) and have type 1 PrP^sc^ (MM1) typically develop rapidly progressive dementia and myoclonus. Pathological examination commonly shows involvement of neocortex, striatum and thalamus, while the hippocampus and brain stem are relatively spared [4]. Individuals who are homozygous for the valine codon (VV) and have type 2 PrP^sc^ (VV2) often present with ataxia and develop dementia at later stages. Pathologically, individuals with VV2 show involvement of the deep neocortical layers, subcortical nuclei and the hippocampal formation [4].

Mitochondrial dysfunction, including mitochondrial respiratory chain (MRC) deficiency and mitochondrial DNA (mtDNA) damage, has been implicated in multiple neurodegenerative diseases [6], but has not been as extensively explored in sCJD. Mitochondrial abnormalities, including morphological alterations, MRC deficiency and impaired mitochondrial dynamics have been reported by studies in cell- and animal models of prion disease [7–10]. Furthermore, one study indicated decreased RNA and protein expression levels for several MRC subunits in human bulk brain tissue [11]. However, since disease-associated changes in cell-type composition have been shown to confound mitochondrial measurements in bulk brain tissue [12, 13], it is unknown to what degree the reported findings reflected MRC depletion in individual cells, or altered cell composition in the CJD samples. While decreased immunofluorescent staining for subunits of complexes I, IV and V was also observed in sCJD [11], this has not been systematically studied. Thus, the question of whether aberrant mitochondrial function occurs within neurons of the sCJD brain remains largely unexplored.

In this work, we study the MRC complexes in single neurons of severely and mildly affected regions in the brain of individuals with either MM1 or VV2 molecular subtypes of sCJD. Our findings show that there is deficiency of all MRC complexes in the temporal cortex of sCJD. Interestingly, MRC deficiency has a predilection for the MM1 molecular subtype of sCJD and is strongly associated with neuropathological markers of the disease.

## Material and methods

### Patient cohorts and tissue samples

Brain tissue from individuals with sCJD (*n* = 20) and controls (*n* = 6) was obtained from the brain collection of the Austrian Creutzfeldt-Jakob Surveillance at the Institute of Neurology, Medical University of Vienna. We chose to study two regions showing different pathological involvement in sCJD: (i) the temporal cortex, which is severely affected, and (ii) the CA4 and CA3 subregions of the hippocampus, which are considered relatively spared of neuropathological changes [14]. Brains were collected at autopsy and sampling was performed as previously described [15]. All samples were collected using a standard technique and a mean fixation time of 30 days. Tissue with suspected prion disease was treated in 98% formic acid (FA) for one hour prior to paraffin embedding according to routine. Our material includes three neurologically healthy controls (Ctrl), and three disease-controls (Ctrl_D_) in which CJD was initially suspected but excluded after neuropathological examination. Ctrl_D_ showed signs of neurodegeneration including senile neurodystrophic changes and chronic vascular lesions, but an exact diagnosis was not available. Ctrl_D_ were included to study the effect of FA treatment on the tissue, as neurologically healthy controls had not been FA treated. Individuals with sCJD were grouped according to codon 129 polymorphism of the *PRNP* gene and PrP^sc^ type (n_MM1_ = 10, n_VV2_ = 10) based on neuropathological examination as previously described [16]. All included individuals were examined by a neuropathologist at autopsy and sections were stained with hematoxylin and eosin using a standard protocol and with antibodies against PrP^sc^ (12F10sc, SPIbio #A03221, dilution 1: 2000). Sections were autoclaved at 121 °C for 10 min following 5 min treatment in concentrated FA and proteinase K. All sections were stained using the Autostainer Link 48 by Agilent Dako. There was no difference in post-mortem delay, formalin fixation time or age between controls, and individuals with MM1- or VV2 molecular subtypes of sCJD. Furthermore, there was no difference in the disease duration between individuals in the MM1 and VV2 groups. Sex was not balanced between the controls (5 females and 1 male) and patients (9 females and 11 males). As previous studies have not shown gender-dependent differences in mitochondrial staining [17], we deemed this acceptable.

### Immunohistochemistry

Immunohistochemistry (IHC) for mitochondrial markers was carried out on formalin-fixed, paraffin embedded sections from the hippocampus and temporal cortex of individuals with sCJD and controls (Ctrl_D_ and Ctrl). Serial sections with a thickness of 3 μm were deparaffinised in xylene and rehydrated in graded ethanol. Antigen retrieval was performed in 1 mM EDTA at pH 8. All sections were stained using Autostainer Link 48 by Agilent Dako. Sections were blocked in peroxidase-blocking solution. Primary antibodies (all from Abcam) against MRC complexes; complex I (anti-NDUFB8, ab110242, dilution 1: 4000), complex II (anti-SDHA, ab14715, dilution 1: 10,000), complex III (anti-UQCRC2, ab14745, dilution 1: 20,000), complex IV (anti-MTCO1, ab14705, dilution 1: 20,000), complex V (anti-ATP5A, ab14748, dilution 1: 20,000), and the mitochondrial membrane marker VDAC1 (anti-VDAC1, ab14734, dilution 1: 20,000) were diluted in EnVision™ FLEX Antibody Diluent and were incubated at room temperature for 30 min. HRP (horseradish peroxidase)-polymer and DAB (3,3′-Diaminobenzidine) chromogen kits were used for visualization. Peroxidase-blocking solution, TBST buffer, mouse-linker, HRP-polymer and DAB chromogen were all from Agilent (EnVision FLEX+, Mouse, High pH (Link), K8002).

### Immunohistochemistry analysis

Both FA treated and -untreated tissue was available from the Ctrl_D_-group, which allowed us to study the effect of FA pretreatment in single individuals. Only the FA treated sections were later included for comparisons between sCJD and the entire control group. To evaluate the effect of FA treatment, tissue from the Ctrl_D_-group that had either been FA treated or not was analysed using a semi-quantitative method to calculate the fraction of positive pixels per neuron as previously described [17]. All sections stained with antibodies against MRC complexes or VDAC1 were analyzed visually and the percentage of positive or deficient neurons was determined in the entire CA4, including the transition into CA3, and in a 2.5 mm^2^ area in the temporal cortex in close relation to the transition from entorhinal cortex into the temporal cortex. Neurons were identified based on morphology, and only neurons where the nucleus and nucleolus were clearly visible were included. All sections were scanned using the NanoZoomer 2.0-HT While Slide Imager (Hamamatsu), and image analyses were conducted using the NDP.view2 (v2.7.25). Neurons from the CA4/CA3 regions were evaluated at 20X magnification, while neurons from the temporal cortex were evaluated at 40X magnification.

### Neuropathological assessment

**Fig. 1:**
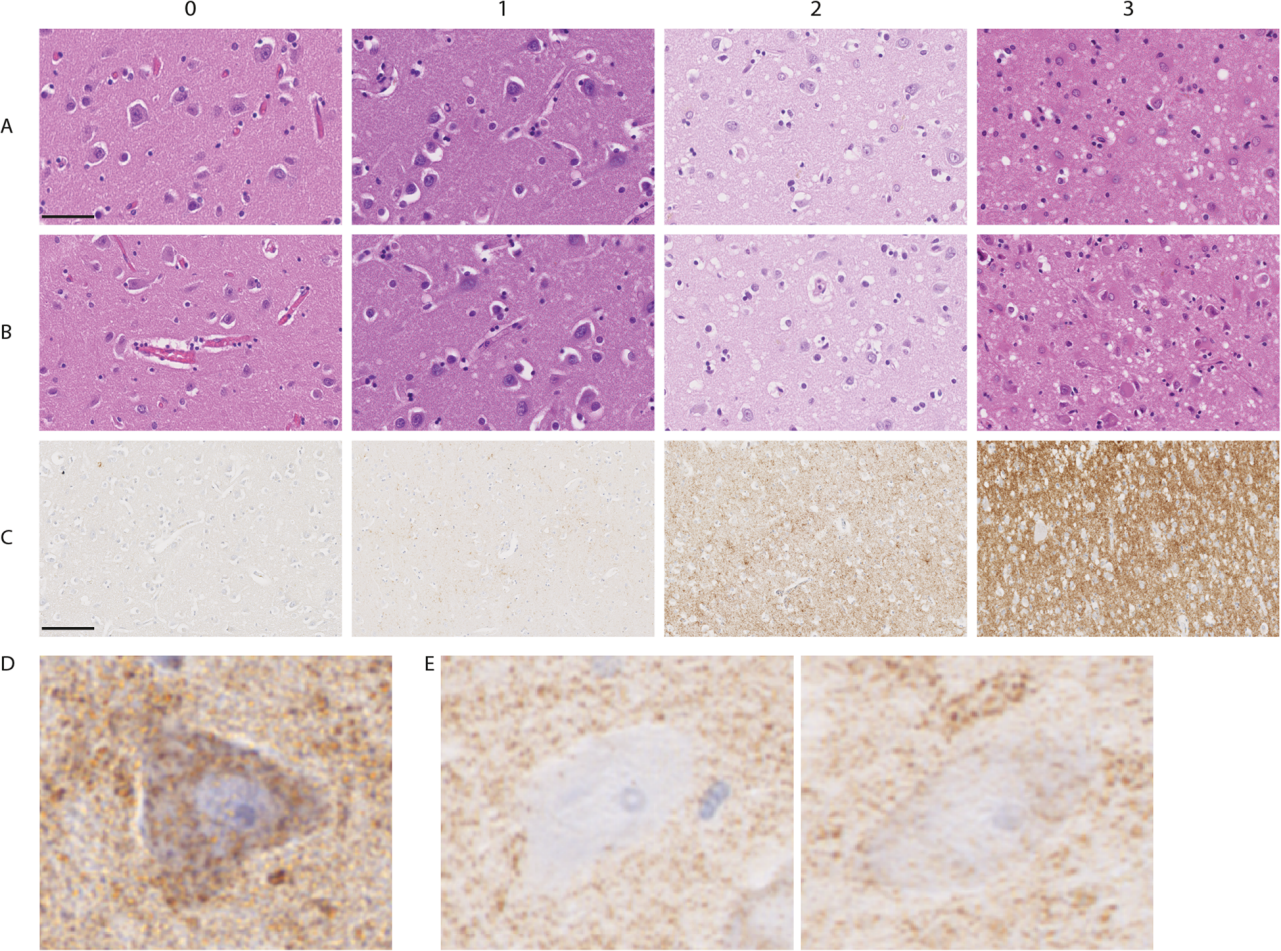
Neuropathological evaluation of sCJD tissue. Representative examples of the neuropathological evaluation of our samples. (A-B) Gliosis (**a**) and vacuolation (**b**) were scored based on hematoxylin and eosin stainings on a scale from no gliosis/vacuolation (0), to slight (1), moderate (2) and severe (3) state. Scalebar: 50 μm. (**c**) Representative examples of PrP^sc^-immunoreactivity in a negative control (0), and individuals with MM1 type sCJD showing slight (1), moderate (2) and prominent (3) diffuse/synaptic immunoreactivity. Scale bar: 100 μm. (**d-e**) Neurons stained with mitochondrial markers were dichotomized and labeled as positive (**d**) or deficient (negative and intermediate staining) (**e**)

Hippocampal sections stained with hematoxylin and eosin (H&E) were scored with regard to gliosis and vacuolation based on a scale from 0 (no astrocytic gliosis/vacuolation) to 3 (severe astrocytic gliosis/vacuolation) as previously reported [5]. Reactive astrocytes were identified from their typically swollen eosinophilic cytoplasm. Only cells with a clearly visible nucleus were evaluated. The neuropathological scoring was performed by two investigators (IHF and GGK) and was done in a region-specific manner resulting in one score from the CA4 region and a separate score from the temporal cortex. In addition, region-specific morphometric analysis of immunoreactivity to PrP^sc^ was calculated using Fiji (v.2.0.0) [18]. For the morphometric analyses, single images from the regions of interest were deconvoluted using the color deconvolution tool and an automatic threshold was set using the Isodata threshold mode. Next, all images were binarized and a fraction of measured pixels per image was used to give a score of percentage of PrP^sc^ stain per image. The Isodata threshold mode was chosen as it was the best fit for strongly and intermediately stained images. Negative images were not analyzed (*n* = 3 in the CA4 of the MM1 group) due to an overestimation of immunoreactivity. Types of PrP^sc^ deposits were not considered in the analyses. However, images from the temporal cortex were acquired in the deeper cortical layers (5 and 6). All images were acquired at 20X magnification. Examples of scores for the neuropathological assessment are shown in Fig. 1.

### Statistical analysis

Statistical analyses were performed in SPSS (v.25.0.0) and Graphpad Prism (v7.0b). Clinical data were compared using t-tests, unless the data were not normally distributed in which case Mann-Whitney U-test was used. Normality was tested using the Shapiro-Wilk test. Equality of variances was tested using Levene’s test. A one-way ANOVA was conducted between the groups (Ctrl, MM1 and VV2) to test differences of means of the MRC complex analyses. Due to the unequal sample sizes, the Welch’s test for unequal variance was used. Eta squared (η^2^) was used to determine effect size of any significant difference. Games-Howell post hoc test was chosen to decide at what level the groups were significantly different due to their unequal sample sizes and variance. The relationship between neuropathology and MRC deficiency was assessed by pair-wise Spearman’s correlations. Due to a small sample size, correlations were studied in the entire sCJD group. A linear regression was performed to evaluate covariation between the molecular subtype of sCJD and vacuolation as a marker of neuropathology. A *P*-value < 0.05 was considered statistically significant in all analyses. Data are presented as mean and 95% confidence interval (CI).

## Results

### Effect of formic acid treatment on immunohistochemistry

**Fig. 2:**
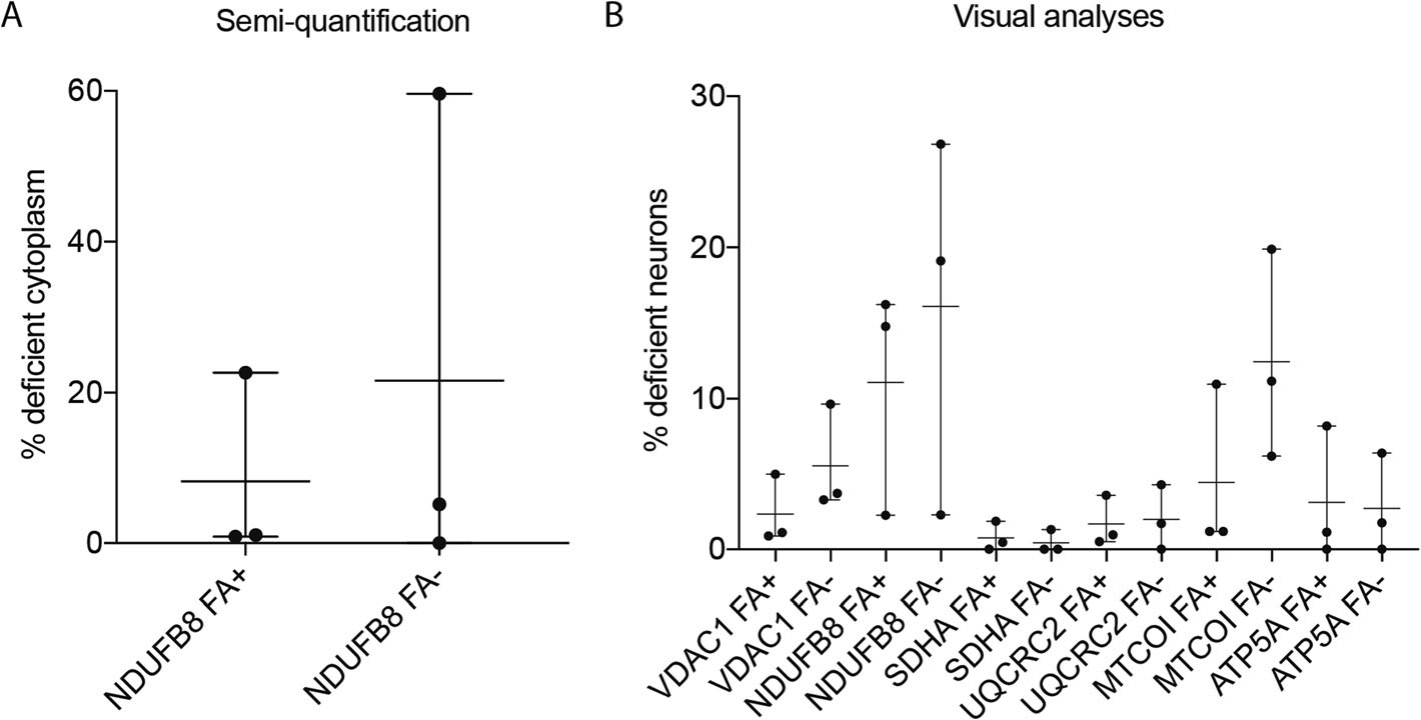
Effect of formic acid treatment on immunohistochemistry. Formic acid treated (FA+) and untreated (FA-) tissue from disease-controls was compared using two different approaches. Findings from analyses in the temporal cortex are shown. **a** Semi-quantification of neuronal cytoplasmic stain, here exemplified by complex I (NDUFB8), shows less MRC deficiency in FA+ compared to FA- from the same three individuals. **b** Visual analysis and categorization of the same sections and individuals resulted in more consistent results. % deficient cytoplasm: mean percentage of neuronal unstained cytoplasm, % deficient neurons: percentage of total neurons from 2.5 mm^2^ of temporal cortex that did not stain positive. Bars show mean and range

Using semi-quantification of unstained cytoplasm, we found that FA treatment slightly skewed the measurements towards less MRC deficiency compared to non-FA treated tissue (Fig. 2a). To mitigate the confounder of FA treatment effects on the tissue analyses, we chose instead a visual binary analysis approach to categorize each neuron as either MRC positive or deficient. This approach produced substantially more consistent results between FA treated and untreated tissues (Fig. 2b). Specifically, FA treatment had no effect on the staining of VDAC1 or MRC complexes II, III and V. For the MRC complexes I and IV FA treatment had a minor effect on staining and was associated with a slight underestimation of deficient neurons in FA-treated tissue. Thus, FA treatment could potentially skew our results towards less deficient neurons for complexes I and IV in sCJD. As a result of this, the reported deficiency of MRC complexes I and IV in sCJD may be slightly underestimated in this work.

### Neuronal MRC deficiency in sCJD

**Fig. 3:**
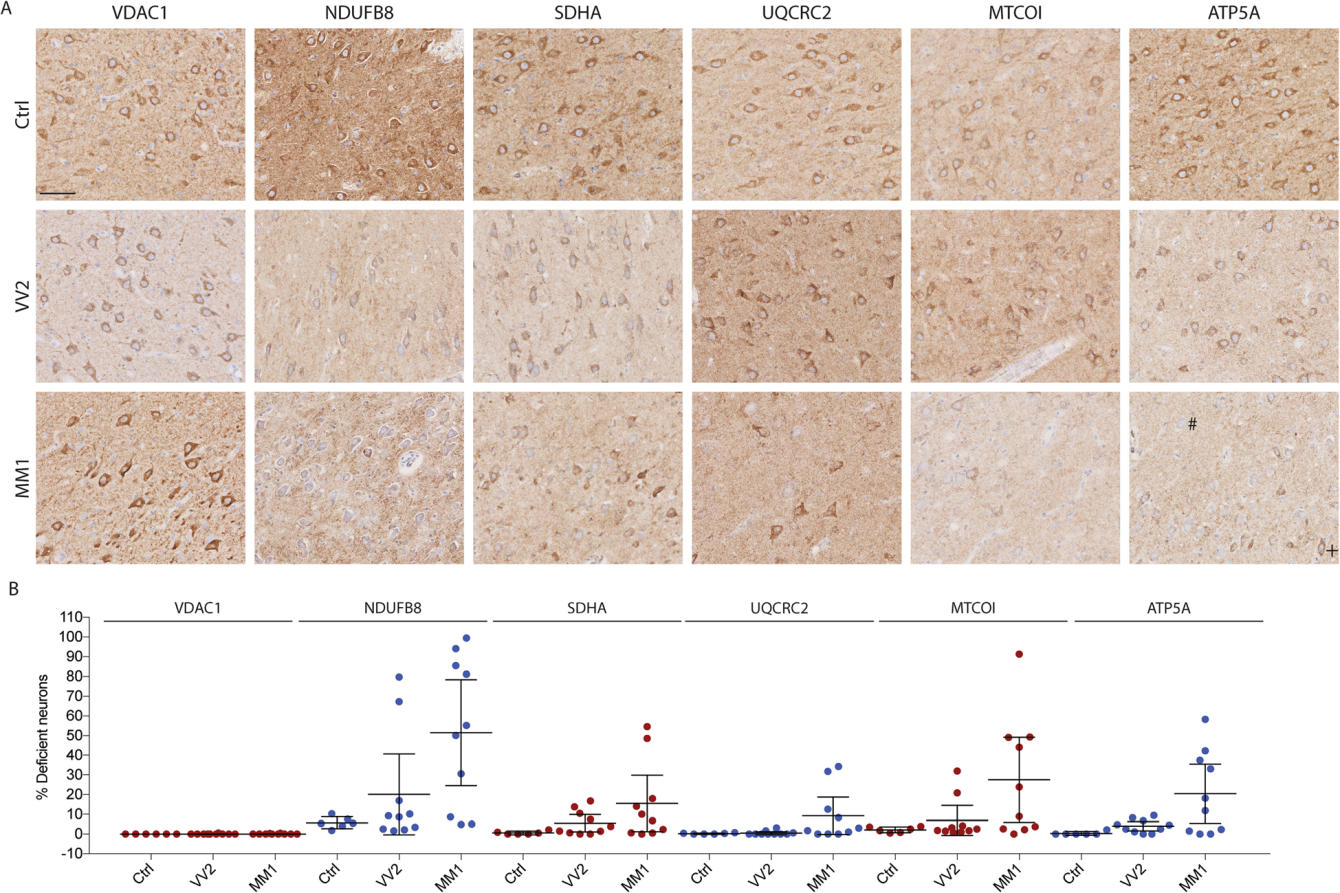
Mitochondrial respiratory chain deficiency in sCJD hippocampus (CA4). **a** Representative photographs of mitochondrial respiratory chain complex stainings (complex I: NDUFB8, complex II: SDHA, complex III: UQCRC2, complex IV: MTCO1, complex V: ATP5A) and VDAC1 in the CA4 region of controls (top row), sCJD molecular subtypes VV2 (middle row) and MM1 (lower row). Mitochondrial respiratory chain deficient neurons are visible in both MM1 and VV2 (+: positive neurons, #: deficient neurons). Decreased staining is observed both in neurons and in surrounding neuropil. Scale bar: 100 μm. **b** Scatter plots showing the fraction of deficient neurons in the CA4 region of the hippocampus. Each dot represents the mean percentage of deficient neurons for each respiratory complex per individual with sCJD (VV2 or MM1) or control. Bars show mean and 95%- confidence intervals**]**

**Fig. 4:**
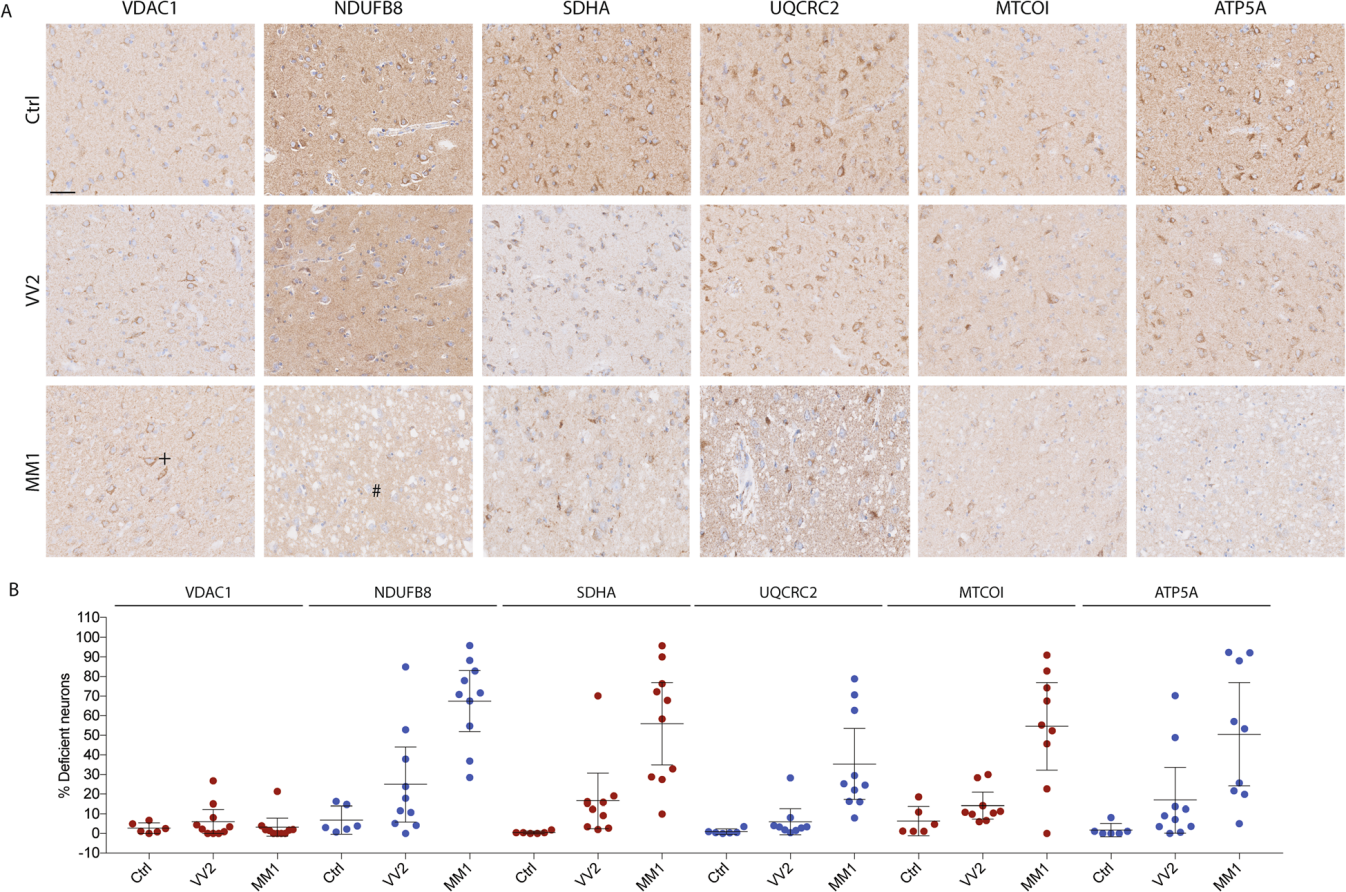
Mitochondrial respiratory chain deficiency in sCJD temporal cortex. **a** Representative photographs of mitochondrial respiratory chain complexes I-V staining (complex I: NDUFB8, complex II: SDHA, complex III: UQCRC2, complex IV: MTCO1, complex V: ATP5A) and VDAC1 in the temporal cortex of controls (top row), sCJD molecular subtypes VV2 (middle row) and MM1 (lower row). Evidence of MRC deficiency is observed in both MM1 and VV2 (+: positive neurons, #: deficient neurons). Scale bar: 50 μm. **b** Scatter plots showing the fraction of deficient neurons in the temporal cortex. Each dot represents the mean percentage of deficient neurons for each respiratory complex per individual with sCJD (VV2 or MM1) or control. Bars show mean and 95% confidence intervals

To assess the MRC in the sCJD-brain, we performed IHC for MRC complexes I-V in the CA4 region (Fig. 3) and temporal cortex (Fig. 4) of sCJD patients and controls. VDAC1 was used as a marker for mitochondrial mass. In the temporal cortex, sCJD patients had a significantly higher fraction of MRC deficient neurons compared to controls. Significant deficiency was found for all MRC complexes (complex I; *P* = 0.001 η^2^ = 0.60, complex II; *P* = 0.001, η^2^ = 0.55, complex III; *P* = 0.003, η^2^ = 0.49, complex IV; *P* = 0.001, η^2^ = 0.73, complex V; *P* = 0.003, η^2^ = 0.40). A similar, but overall less pronounced deficiency was found in the CA4/CA3 region (complex I; *P* = 0.006 η^2^ = 0.31, complex II; *P* = 0.019, η^2^ = 0.20, complex III; *P* = 0.119, complex IV; *P* = 0.040, η^2^ = 0.26, complex V; *P* = 0.003, η^2^ = 0.33; Table 2). VDAC1 staining revealed no difference in mitochondrial mass between groups in either region (temporal cortex; *P* = 0.542, CA4/CA3; *P* = 0.621). The total number of evaluated neurons in each group, means with 95% CI and results of one way-ANOVA are summarized in Table 2. As reflected in their means and 95% CI’s, there was no difference in staining intensity of neither mitochondrial marker nor MRC complexes between Ctrl_D_ and Ctrl. Subject demographics of included individuals are summarised in Table 1.

**Table 1:** Demographic overview, post-mortem delays and fixation times of included samples

ID	AOD	Sex	Molecular subtype	DD (months)	PMD (h)	FT (days)
**sCJD**						
sCJD1	62	F	VV2	4	72	10
sCJD2	58	F	VV2	7	24	46
sCJD3	61	M	VV2	3	24	19
sCJD4	73	F	VV2	4	48	31
sCJD5	69	M	VV2	4	72	22
sCJD6	75	M	VV2	6	24	18
sCJD7	62	M	VV2	2	48	14
sCJD8	65	M	VV2	6	72	21
sCJD9	75	M	VV2	2	24	24
sCJD10	75	M	VV2	8	24	17
sCJD11	80	F	MM1	4	24	27
sCJD12	66	F	MM1	6	96	3
sCJD13	74	F	MM1	4	48	32
sCJD14	68	F	MM1	3	24	44
sCJD15	72	F	MM1	3	24	56
sCJD16	67	M	MM1	6	48	112
sCJD17	69	M	MM1	2	24	15
sCJD18	72	M	MM1	6	< 24	16
sCJD19	80	F	MM1	1.5	48	98
sCJD20	70	M	MM1	8	24	30
Ctrl_D_						
Ctrl1	67	F	–	–	24	31
Ctrl2	78	F	–	–	48	14
Ctrl3	81	F	–	–	24	37
Ctrl						
Ctrl4	65	F	–	–	24	20
Ctrl5	73	F	–	–	48	22
Ctrl6	81	M	–	–	24	15

*CJD* Sporadic Creutzfeldt-Jakob disease, *Ctrl*_*D*_ Disease-control, *Ctrl* Control, *AOD* Age of death, *PMD* Post-mortem delay (hours), *FT* Fixation time (days), *F* Female, *M* Male, *DD* Disease duration in months

### MRC deficiency is more pronounced in the MM1 molecular subtype of sCJD

Individual assessment of MM1- and VV2 sCJD molecular subtypes showed highly significant differences in terms of MRC deficiency. Compared to controls, tissue from MM1 molecular subtype of sCJD exhibited significant deficiency of all MRC complexes in the temporal cortex (complex I; *P* <  0.001, complex II; *P* = 0.001, complex III; *P* = 0.005, complex IV; *P* <  0.001, complex V; *P* = 0.007) and of complex I (*P* = 0.010) and complex V (*P* = 0.034) in the CA4/CA3 region. MRC staining was substantially less affected in VV2 molecular subtype of sCJD. With the exception of mild complex V (*P* = 0.019) deficiency in the CA4/CA3 region, neuronal MRC staining in the VV2 molecular subtype of sCJD was not significantly different from controls (Table 2).

**Table 2:** Visual analyses of mitochondrial respiratory chain staining

	Protein	Group	Number of Neurons	% Deficient neurons				
				Mean (95% CI)	ANOVA Welch (***P*** =)	η^2^	Group comparisons	GH (***P*** =)
**CA4/CA3**	VDAC1	Ctrl	1242	0 (0,0)	0.621	–	Ctrl vs VV2	–
		VV2	2404	0.06 (−0.07,0.19)			Ctrl vs MM1	–
		MM1	2411	0.08 (−0.04,0.19)			MM1 vs VV2	–
	NDUFB8	Ctrl	1442	5.76 (2.72, 8.80)	**0.006**	0.310	Ctrl vs VV2	0.302
		VV2	2499	20.17 (− 0.32, 40.66)			Ctrl vs MM1	**0.010**
		MM1	2085	51.45 (24.51, 78.39)			MM1 vs VV2	0.122
	SDHA	Ctrl	1194	0.61 (−0.30, 1.51)	0.019	0.195	Ctrl vs VV2	0.075
		VV2	2052	5.58 (1.17, 9.99)			Ctrl vs MM1	0.097
		MM1	1878	15.55 (1.27, 29.83)			MM1 vs VV2	0.325
	UQCRC2	Ctrl	1381	0.26 (−0.07, 0.59)	0.119	–	Ctrl vs VV2	–
		VV2	1675	0.54 (−0.21, 1.29)			Ctrl vs MM1	–
		MM1	1913	9.28 (−0.14, 18.71)			MM1 vs VV2	–
	MTCO1	Ctrl	1282	2.18 (0.71, 3.64)	**0.040**	0.255	Ctrl vs VV2	0.373
		VV2	2263	6.99 (−0.61, 14.58)			Ctrl vs MM1	0.062
		MM1	2000	27.52 (5.92, 49.12)			MM1 vs VV2	0.151
	ATP5A	Ctrl	1252	0.37 (−0.57, 1.30)	**0.003**	0.331	Ctrl vs VV2	**0.019**
		VV2	1803	4.01 (1.62, 6.40)			Ctrl vs MM1	**0.034**
		MM1	1554	20.96 (5.49,35.48)			MM1 vs VV2	0.082
**Temporal cortex**	VDAC1	Ctrl	1942	2.71 (−0.05, 5.46)	0.542	–	Ctrl vs VV2	–
		VV2	2876	6.10 (−0.09, 12.29)			Ctrl vs MM1	–
		MM1	2784	3.26 (−1.38, 7.89)			MM1 vs VV2	–
	NDUFB8	Ctrl	1275	6.82 (−0.31, 13.96)	**0.001**	0.603	Ctrl vs VV2	0.116
		VV2	2116	27.68 (7.12, 48.25)			Ctrl vs MM1	**< 0.001**
		MM1	1633	67.50 (51.98, 83.01)			MM1 vs VV2	**0.008**
	SDHA	Ctrl	1171	0.54 (−0.18, 1.25)	**0.001**	0.547	Ctrl vs VV2	0.071
		VV2	1822	16.62 (2.47, 30.77)			Ctrl vs MM1	**0.001**
		MM1	1107	55.95 (34.93, 76.97)			MM1 vs VV2	**0.008**
	UQCRC2	Ctrl	1461	0.97 (−0.42, 2.36)	**0.003**	0.492	Ctrl vs VV2	0.278
		VV2	1666	5.38 (−0.60, 11.37)			Ctrl vs MM1	**0.005**
		MM1	975	35.39 (17.23, 53.56)			MM1 vs VV2	**0.012**
	MTCO1	Ctrl	1237	6.31 (−1.12, 13.75)	**0.001**	0.727	Ctrl vs VV2	0.179
		VV2	1895	14.18 (7.38, 20.97)			Ctrl vs MM1	**< 0.001**
		MM1	1515	58.60 (41.42, 75.77)			MM1 vs VV2	**0.001**
	ATP5A	Ctrl	1758	1.78 (−1.57, 5.13)	**0.003**	0.404	Ctrl vs VV2	0.162
		VV2	2034	16.92 (0.14, 33.70)			Ctrl vs MM1	**0.007**
		MM1	1127	50.57 (24.19, 76.94)			MM1 vs VV2	0.066

The table summarizes results of one-way ANOVA and eta squared analysis of effect where a significant difference at the group level. 95% CI: 95% confidence interval, η^2^; eta squared, GH: Games-Howell, Ctrl: control, MM1: MM1 molecular subtype of sCJD, VV2; VV2 molecular subtype of sCJD. Statistically significant *P*-values are in bold type

### Complex I deficiency in the temporal cortex correlates with sCJD neuropathology

**Fig. 5:**
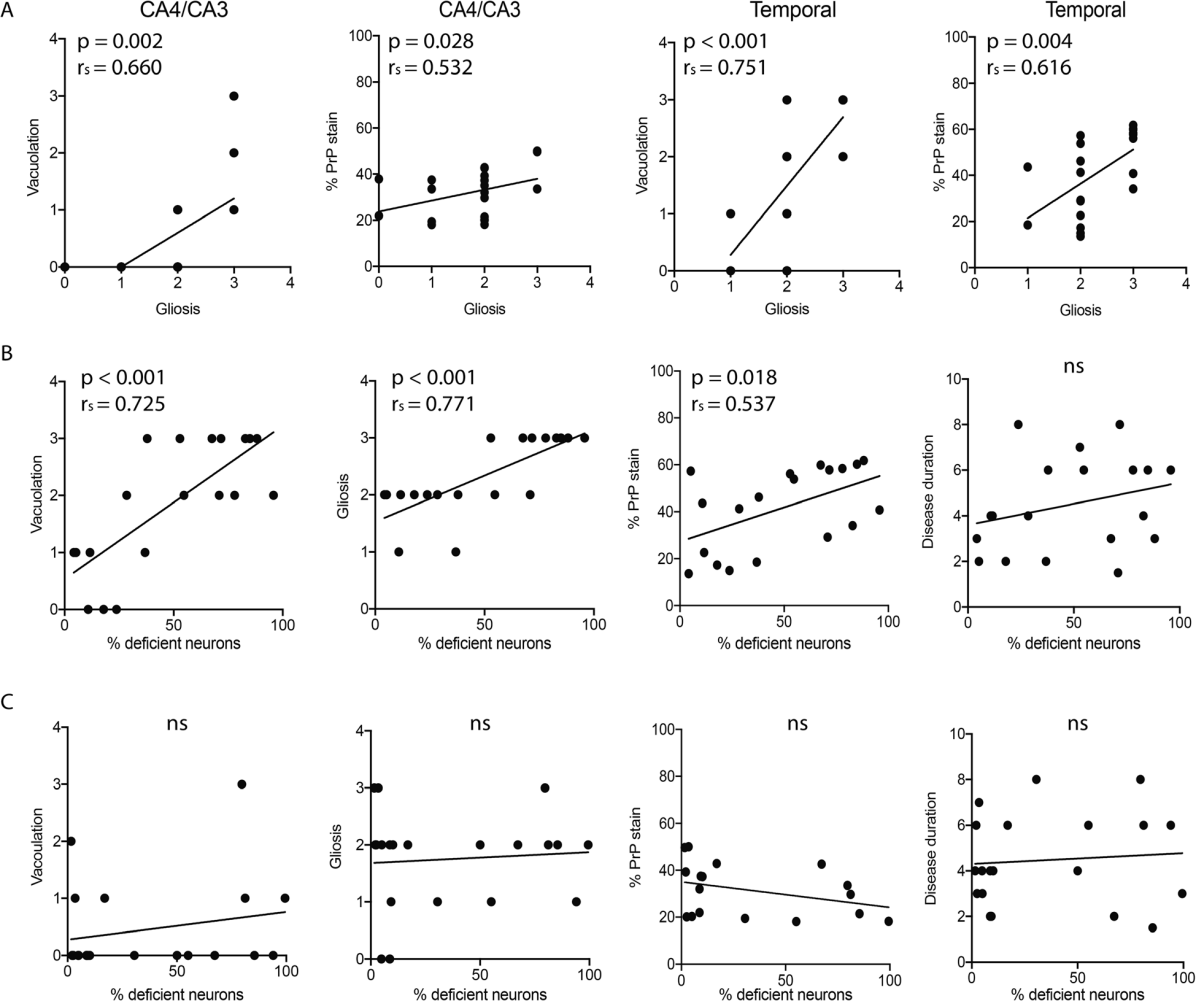
Neuropathology correlates with complex I deficiency in the temporal cortex. Correlation plots of clinicopathological variables and MRC deficiency, exemplified by complex I (NDUFB8). Vacuolation and PrP^sc^-immunoreactivity correlate with gliosis in CA4/CA3 and temporal cortex (**a**). Vacuolation, gliosis and PrP^sc^-immunoreactivity correlate with mitochondrial complex I deficiency in the temporal cortex, (**b**) but not the hippocampal regions of CA4/CA3 (**c**)

Next, we sought to investigate whether the MRC deficiency correlated with neuropathology markers of sCJD (Fig. 5). To this end, we chose complex I deficiency as a representative marker of MRC deficiency. Individual scores of neuropathology markers and means with 95% CI are listed in Table 3. As expected, vacuolation and PrP^sc^-staining correlated significantly with gliosis in all examined areas (Fig. 5a). Intriguingly, in the temporal cortex, we found a significant positive correlation between the amount of complex I deficient neurons and vacuolation (*P* <  0.001, r_s_ = 0.725), gliosis (*P* <  0.001, r_s_ = 0.771) and PrP^sc^ stain (*P* = 0.018, r_s_ = 0.537; Fig. 5b). In contrast, the hippocampal regions CA4/CA3 showed no correlation with any of the neuropathology markers (vacuolation; *P* = 0.699, gliosis; *P* = 0.605, PrP^sc^ stain; *P* = 0.109; Fig. 5c). Disease duration did not correlate with the percentage of complex I deficient neurons in either temporal cortex (*P* = 0.227) or the CA4/CA3 hippocampal regions (*P* = 0.951). Linear regression with vacuolation as covariate confirmed that molecular subtype of sCJD is a predictor of MRC deficiency (Beta = 0.40, *P* = 0.019), however, this difference could be partly confounded by severity of vacuolation (Beta = 0.57, *P* = 0.002).

**Table 3:** Neuropathological assessments

		CA4/CA3			Temporal		
ID	Type	% PrP stain	Gliosis	Vacuo	% PrP stain	Gliosis	Vacuo
**sCJD**							
sCJD1	VV2	37.26	2	0	22.64	2	1
sCJD2	VV2	50.04	3	1	56.09	3	3
sCJD3	VV2	20.15	2	0	13.65	2	1
sCJD4	VV2	49.70	3	2	28.90	2	2
sCJD5	VV2	32.07	2	0	43.63	1	0
sCJD6	VV2	39.31	2	0	46.17	2	3
sCJD7	VV2	42.61	2	0	17.31	2	0
sCJD8	VV2	42.88	2	1	60.23	3	3
sCJD9	VV2	37.42	1	0	57.27	2	1
sCJD10	VV2	33.55	3	3	14.93	2	0
**Mean (95% CI)**		38.50 (32.19, 44.81)	2.20 (1.75, 2.65)	0.7 (−0.06, 1.46)	36.08 (22.75, 49.41)	2.10 (1.69, 2.51)	1.4 (0.50, 2.30)
sCJD11	MM1	–	2	0	34.13	3	3
sCJD12	MM1	18.16	1	0	53.85	2	2
sCJD13	MM1	–	0	0	41.26	2	2
sCJD14	MM1	18.17	2	1	61.81	3	3
sCJD15	MM1	20.25	2	0	59.89	3	3
sCJD16	MM1	–	1	0	40.80	3	2
sCJD17	MM1	21.99	0	0	18.50	1	1
sCJD18	MM1	29.68	2	1	58.40	3	2
sCJD19	MM1	21.48	2	0	29.17	2	2
sCJD20	MM1	19.43	1	0	57.80	3	3
**Mean (95% CI)**		21.31 (17.63, 24.99)	1.43 (0.70, 2.16)	0.29 (−0.17, 0.74)	48.49 (32.50, 64.48)	2.43 (1.70, 3.16)	2.29 (1.59, 2.98)
**Ctrl**_**D**_							
Ctrl1	**–**	–	1	0	–	1	0
Ctrl2	**–**	–	1	0	–	0	0
Ctrl3	**–**	–	0	0	–	0	0
**Ctrl**							
Ctrl4	–	–	0	0	–	0	0
Ctrl5	–	–	0	0	–	1	0
Ctrl6	–	–	2	0	–	1	0

sCJD = sporadic Creutzfeldt-Jakob disease, Ctrl_D_ Diseased control, *Ctrl* Control, *MM1* MM1 molecular subtype sCJD, *VV2* VV2 molecular subtype sCJD, % PrP stain = % morphometric score of prion stain, Gliosis = score gliosis, Vacuo = score vacuolation

## Discussion

We show that neuronal MRC deficiency affecting all respiratory complexes occurs in the brain of patients with sCJD. This deficiency shows a strong association with the severity of pathological markers of disease and has a predilection for the MM1 molecular disease subtype. In spite of the pronounced MRC loss, the outer mitochondrial membrane marker VDAC1 remains intact, indicating that the total neuronal mitochondrial mass remains unchanged. The finding of deficient MRC in sCJD corroborate the work of Ansoleaga et al., who reported deficiency of multiple MRC complexes in the frontal cortex of MM1 type sCJD [11].

Neuronal MRC deficiency in sCJD shows a strong correlation with the severity of the neurodegenerative changes. It has a clear regional predilection for the severely affected temporal cortex, whereas the relatively spared CA4/CA3 hippocampal regions show only a mild decrease in MRC staining. Moreover, the extent of MRC deficiency in the temporal cortex shows a positive correlation with the severity of neuropathological changes including gliosis, vacuolation and PrP^sc^ load. We believe that the lack of association between MRC deficiency and severity of neuropathology in the CA4/CA3 region may reflect insufficient statistical power due to the substantially milder MRC involvement in that area.

The severity of MRC deficiency is associated with the molecular subtype of sCJD. MRC deficiency is significantly more pronounced in neurons of individuals with MM1 type sCJD compared to VV2 type sCJD. In fact, with the exception of complex V, MRC deficiency in VV2 type sCJD was not statistically significant compared to controls, although a trend was seen for higher numbers of MRC-deficient neurons. Regression analysis shows that the difference between molecular subtype of sCJD is partly confounded by the more severe pathological changes in MM1 molecular phenotype. Spongiform changes are more pronounced in the neocortex of MM1 type sCJD compared to VV2, where the basal ganglia and thalamus are predominantly affected [19]. However, as disease subtype remains a significant predictor of MRC deficiency in our model, we cannot exclude the possibility of additional factors rendering MM1 neurons more susceptible to MRC impairment.

Our findings suggest that MRC deficiency is an integral part of sCJD pathology and plays an important role in the pathogenesis of the disorder. While MRC deficiency correlates strongly with the severity of disease specific pathological markers, the fact that it is also present in morphologically normal neurons of the mildly affected CA4/CA3 region suggests it is not a terminal phenomenon in dying neuronal populations. The mechanisms underlying MRC loss in sCJD remain unknown. Since mitochondrial mass is unchanged, it is possible MRC is depleted via an active downregulation or increased degradation of the peptide subunits of the respiratory complexes. mtDNA encodes subunits of complexes I, III, IV and V and quantitative and/or qualitative mtDNA defects are a common cause of MRC deficiency [20]. However, mtDNA defects should not affect complex II, which is entirely encoded in the nucleus, and very rarely cause a uniform loss of the remaining respiratory complexes. Therefore, the MRC deficiency of sCJD does not fit the profile of pure mtDNA defects.

The observed pan-respiratory complex deficiency without a change in the mitochondrial outer membrane marker VDAC1 may reflect damage to the inner mitochondrial membrane. This is in line with previous reports of abnormal mitochondrial cristae architecture in prion infected rodents [8, 21, 22]. Interestingly, while PrP^c^ has been shown to localize mostly on the plasma membrane, it has been suggested that it may also be present in the inner mitochondrial membrane of healthy mice [23]. Furthermore, dysmorphic mitochondria with abnormal cristae morphology have been observed in *PRNP* knockout mice [24]. It is therefore possible that PrP^sc^ aggregation injures the inner mitochondrial membrane, both directly and via loss of its physiological counterpart, PrP^c^.

Irrespective of the cause, deficiency of the entire MRC is expected to compromise neuronal metabolism causing ATP depletion and a shift toward increased glycolysis [25]. Neurons are highly dependent on oxidative phosphorylation and use most of their ATP to maintain their membrane potential. This is achieved via the action of the sodium-potassium ATPase, which maintains the intra- and extracellular concentrations of sodium and potassium ions against their electrochemical gradients and, by extension, regulates intracellular water balance [26]. Pump failure due to ATP deficiency causes a shift of water from the extracellular to the intracellular compartment (i.e. cytotoxic edema), leading to disruption of essential cellular processes and ultimately cell death. In fact, diffusion weighted imaging (DWI) of the brain typically shows evidence of restricted water diffusion in affected areas of the sCJD brain [27], suggesting that cytotoxic edema is indeed an important mechanism underlying neuronal death in sCJD. Based on our findings, we propose that cytotoxic changes in the sCJD brain are caused by ATP depletion due to neuronal MRC deficiency. Similar signs of restricted water diffusion on MRI are seen in other disorders involving neuronal energy failure such as mitochondrial disease [28–30], hypoxia/ischemia [31], hypoglycaemia [32, 33] and carbon monoxide intoxication [34].

Another consequence of neuronal MRC impairment in sCJD would be a shift toward glycolytic metabolism, resulting in higher production of lactate. Elevated concentration of cerebrospinal fluid (CSF) lactate has indeed been reported in patients with CJD, corroborating this hypothesis [35]. In addition to decreased ATP production, a glycolytic shift could render neurons more susceptible to oxidative damage. Normally, neurons can consume glucose through the pentose phosphate pathway, which helps to regenerate reduced glutathione, an important component against oxidative stress. Up-regulation of glycolysis may therefore cause increased levels of oxidized glutathione, in turn, increased susceptibility to the formation of reactive oxygen species in neurons [36]. Oxidative damage has indeed been implicated in cell models of prion disease [37–39].

While cause and effect cannot be confidently discerned by this type of study, our findings strongly suggest that MRC loss in sCJD is deleterious and actively involved in the neurodegenerative process. Interestingly, the pattern of MRC deficiency in sCJD is distinct from that of other neurodegenerative proteinopathies. In Parkinson’s disease, MRC deficiency is selective for complex I (and to a much lesser degree complex IV) and does not correlate with the severity of neurodegeneration. In fact, unlike sCJD where the load of PrP^sc^ correlates with MRC deficiency, in PD there is an inverse relationship between complex I loss and Lewy pathology leading to the hypothesis that it may be a partly protective event [17, 40]. It is conceivable that downregulation of complex I levels without a decrease in the remaining respiratory complexes, as observed in PD, serves to limit reactive oxygen species (ROS) production and oxidative damage without a major compromise in energy transduction. In contrast, the global MRC loss seen in sCJD is highly likely to cause ATP depletion.

In conclusion, we show that mitochondrial dysfunction is an important mechanism underlying neuronal injury and neurodegeneration in sCJD. These findings provide an explanation for the clinical observations of restricted MRI diffusion and elevated CSF lactate in patients. Our findings suggest that mitochondria should be the focus of further study in sCJD and should be assessed as potential therapeutic targets for this incurable and devastating disorder.

## Data Availability

All data used and/or analysed during the current study available from the corresponding author on reasonable request.

## Abbreviations

sCJD: Sporadic creutzfeldt-jakob disease
PrP^sc^: Disease associated prion protein
PrP^c^: Naturally occurring prion protein
MRC: Mitochondrial respiratory chain
mtDNA: Mitochondrial DNA
PrP^c^: Prion protein
FA: Formic acid
Ctrl: Control
Ctrl_D_: Disease-control
CJD: Creutzfeldt-Jakob disease
MRI: Magnetic resonance imaging
IHC: Immunohistochemistry
HRP: Horseradish peroxidase
DAB: 3,3′-Diaminobenzidine
CI: Confidence interval
DWI: Diffusion weighted imaging
CSF: Cerebrospinal fluid
ROS: Reactive oxygen species
